# Protease resistance of porcine acidic mammalian chitinase under gastrointestinal conditions implies that chitin-containing organisms can be sustainable dietary resources

**DOI:** 10.1038/s41598-017-13526-6

**Published:** 2017-10-11

**Authors:** Eri Tabata, Akinori Kashimura, Satoshi Wakita, Misa Ohno, Masayoshi Sakaguchi, Yasusato Sugahara, Yasutada Imamura, Shiro Seki, Hitoshi Ueda, Vaclav Matoska, Peter O. Bauer, Fumitaka Oyama

**Affiliations:** 1 Department of Chemistry and Life Science, Kogakuin University, Hachioji, Tokyo, 192-0015 Japan; 2 Department of Environmental Chemistry, Kogakuin University, Hachioji, Tokyo, 192-0015 Japan; 3 Department of Integrative Biology, Graduate School of Natural Science and Technology, Okayama University, Okayama, 700-8530 Japan; 4 Laboratory of Molecular Diagnostics, Department of Clinical Biochemistry, Hematology and Immunology, Homolka Hospital, Roentgenova 37/2, Prague, 150 00 Czech Republic; 5 Bioinova Ltd., Videnska 1083, Prague, 142 20 Czech Republic

## Abstract

Chitin, a polymer of *N*-acetyl-D-glucosamine (GlcNAc), is a major structural component in chitin-containing organism including crustaceans, insects and fungi. Mammals express two chitinases, chitotriosidase (Chit1) and acidic mammalian chitinase (AMCase). Here, we report that pig AMCase is stable in the presence of other digestive proteases and functions as chitinolytic enzyme under the gastrointestinal conditions. Quantification of chitinases expression in pig tissues using quantitative real-time PCR showed that Chit1 mRNA was highly expressed in eyes, whereas the AMCase mRNA was predominantly expressed in stomach at even higher levels than the housekeeping genes. AMCase purified from pig stomach has highest activity at pH of around 2–4 and remains active at up to pH 7.0. It was resistant to robust proteolytic activities of pepsin at pH 2.0 and trypsin and chymotrypsin at pH 7.6. AMCase degraded polymeric chitin substrates including mealworm shells to GlcNAc dimers. Furthermore, we visualized chitin digestion of fly wings by endogenous AMCase and pepsin in stomach extract. Thus, pig AMCase can function as a protease resistant chitin digestive enzyme at broad pH range present in stomach as well as in the intestine. These results indicate that chitin-containing organisms may be a sustainable feed ingredient in pig diet.

## Introduction

Pigs are an ideal biomedical model for human diseases and conditions bridging the gaps between mouse models and humans^1^. Pigs are more similar to humans in terms of genetics, digestive physiology^2,3^ and metabolism^4^ than mouse, and potentially, they could be used as a source of organs for human transplantations in the future^5,6^. Besides the biomedical purpose, pork is also an important dietary source for humans, accounting for more than half of the world’s meat consumption. Recently, its demands are increasing due to growing human population^7,8^.

Chitin, a linear polymer of β-1, 4-linked *N*-acetyl-D-glucosamine (GlcNAc), is the second most abundant natural polysaccharide in nature and functions as a major structural component in fungi, crustaceans, and insects^9,10^. Although mammals do not produce chitin, humans and mice express two active chitinases which belong to the family 18 of glycoside hydrolases^10,11^. Firstly, chitotriosidase (Chit1) was identified in macrophages of Gaucher disease patients^12–14^. AMCase was discovered later and was named for its acidic isoelectric point^15^. These mammalian chitinases have been considered as a protection against chitin-containing pathogens^10,16^.

Since AMCase expression is significantly altered under several pathological conditions such as asthma, allergic inflammation, ocular allergy, dry eye syndrome and gastric cancer^17–23^, it has attracted considerable attention. Some polymorphisms and haplotypes in the AMCase gene are associated with bronchial asthma in humans^24–26^. Recently, AMCase was shown to be a constitutively produced enzyme essential for chitin degradation in the airways to maintain lung functions^27,28^. In addition, AMCase plays role in the protective immune response to gastrointestinal nematodes in the host gastrointestinal tract (GIT)^29^.

Murine AMCase is most active at pH of around 2.0^15,30,31^. Mouse stomach produces enormous quantities of AMCase mRNA and protein^32,33^. Thus, AMCase seems to function as a digestive enzyme that breaks down chitin-containing ingested material^15,30–32^. Since chitin has long been thought to be not degraded in the mammalian digestive system, it is sometimes included in animal feeds as dietary fiber^34^. Recently we showed that mouse AMCase and chicken Chia, a homologue of AMCase, can function as a protease-resistant major glycosidase under stomach and intestine conditions while degrading chitin substrates to GlcNAc dimer [(GlcNAc)_2_], a source of carbon, nitrogen and energy^35,36^. However, the physiological roles of the AMCase in other mammals remain unknown.

Here, we quantified expression levels of the chitinases in pig tissues by quantitative real-time PCR (qPCR). Also, we purified AMCase from pig stomach tissue and characterized its optimal condition and protease-resistance. We provide evidence that chitin-containing organisms can be digested under pig GIT condition which is supported by degradation products analysis and morphological analysis.

## Results

### Gene expression analysis of Chit1 and AMCase mRNAs in pig tissues

To study the *in vivo* regulation of pig Chit1 and AMCase genes expression, total RNA from various normal pig tissues was analyzed using a qPCR assay with a specifically designed standard DNA (Supplementary Fig. S1) as described in Methods.

**Figure 1:**
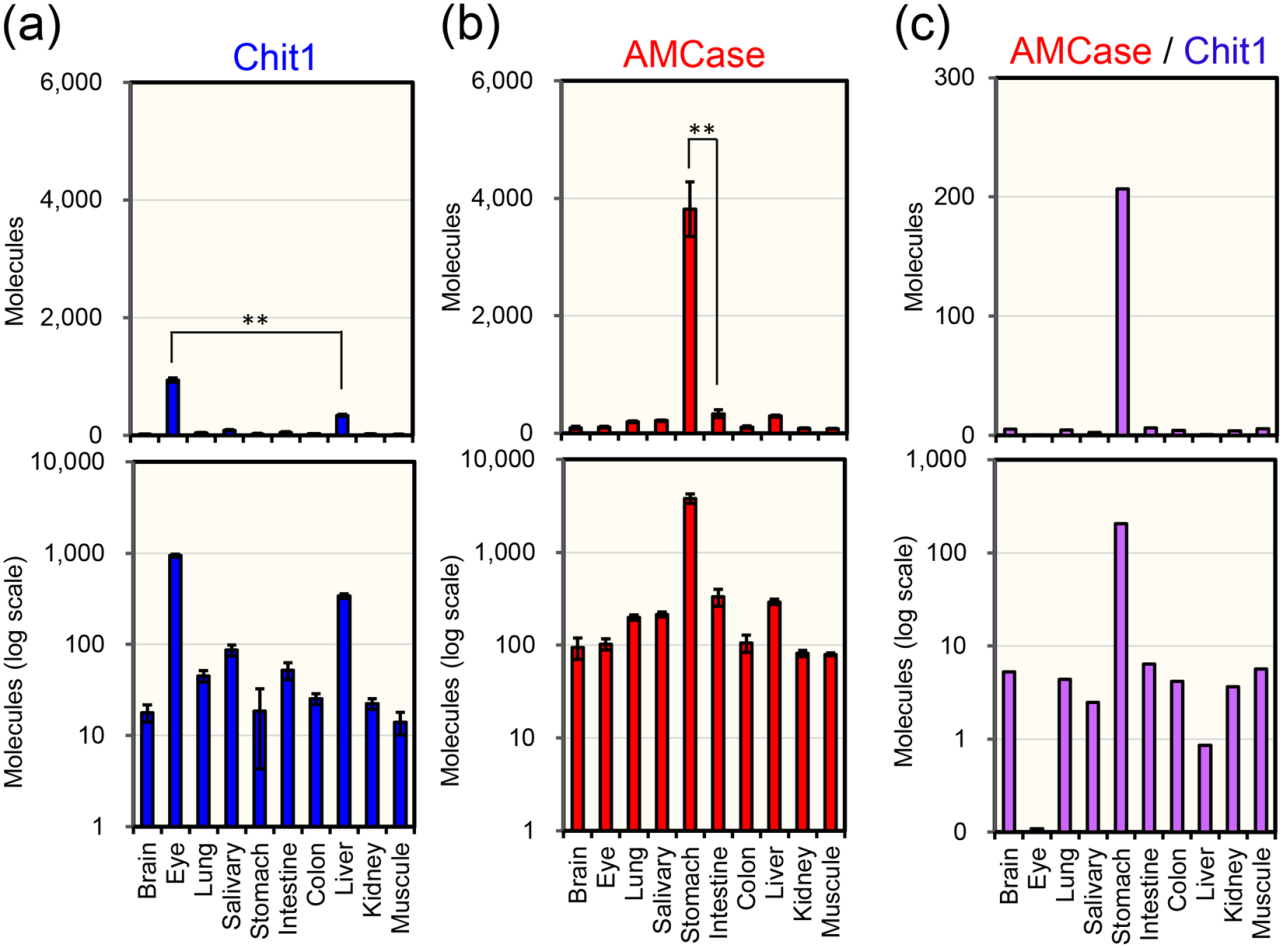
Expression of Chit1 and AMCase mRNAs in pig tissues. Quantification of (**a**) Chit1 and (**b**) AMCase mRNAs in ten major pig tissues. Both chitinases were quantified by qPCR using the standard DNA. All values obtained were expressed as molecules per 10 ng of total RNA. (**c**) Ratios of AMCase to Chit1. All mRNA copy numbers were derived based on the same standard dilutions. The upper panels indicate the actual number, whereas the lower panel shows each value on logarithmic scale. Each reaction was performed in triplicate. **p < *0.01*. P-values were determined using Student’s t-test.

Clear tissue-specific pattern was observed in both chitinases mRNAs expression (Fig. 1a and b, upper panels). High levels of Chit1 mRNA were detected in the eye (Fig. 1a, upper panel), followed by liver, salivary gland, intestine and lung (Fig. 1a, lower panel). AMCase mRNA was predominantly detected in the stomach (Fig. 1b, upper panel), followed by intestine, liver, salivary gland and lung (Fig. 1b, lower panel). The levels of AMCase in all tissues, and particularly in stomach were markedly higher than those of Chit1 (Fig. 1c, upper panel) except for the eye (Fig. 1c, lower panel).

### AMCase mRNA levels in pig stomach

**Figure 2:**
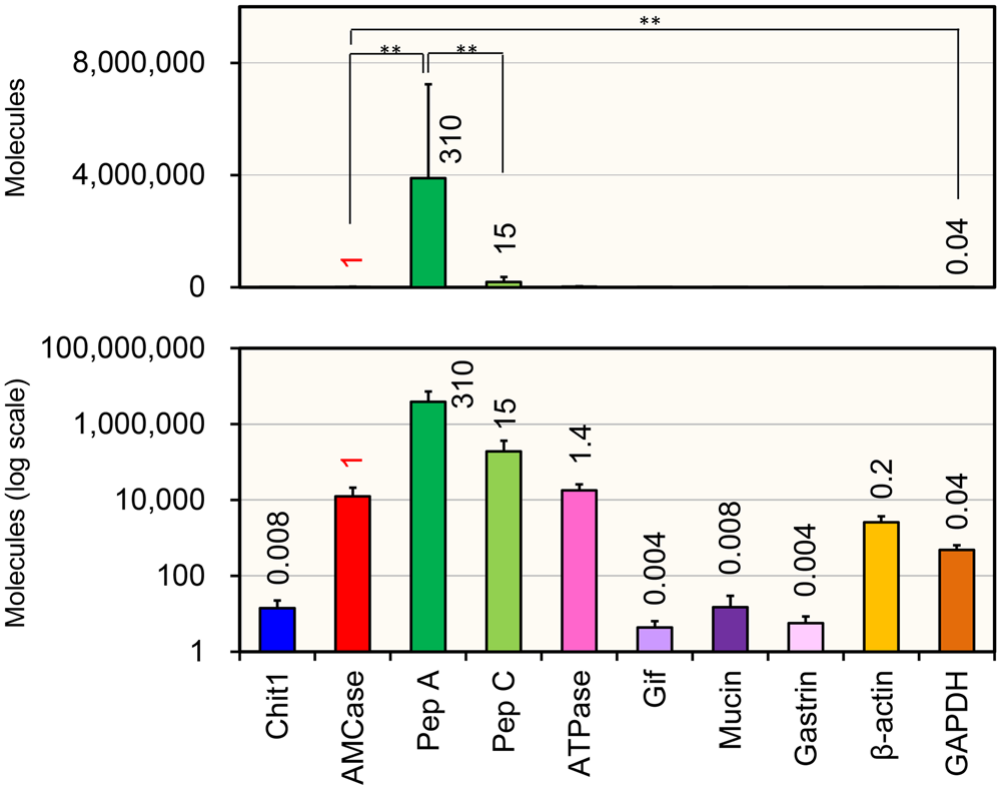
Quantification of mRNA expressions in pig stomach by real-time PCR. Evaluation of AMCase mRNA levels in pig stomach tissues using standard DNA containing 10 genes fragments: Chit1, AMCase, pepsinogen A (Pep A), pepsinogen C (Pep C), H + /K + -ATPase, Gastric intrinsic factor (Gif), mucin, Gastrin, β-actin and GAPDH. The expression levels of the 10 genes determined using the cDNAs prepared from stomach tissues from 6-month-old pig (n = 3) were quantified by qPCR. Expression level of the AMCase gene was set to 1 (written in red); the values on the bars indicate the relative expression levels compared to the expression level of AMCase. The upper panels indicate the actual number, whereas the lower panel shows each value on logarithmic scale. Each reaction was performed in triplicate. **p < 0.01. P-values were determined using Student’s t-test.

Next, we evaluated the AMCase mRNA levels in pig stomach in detail. The mRNAs of pepsinogen A and C, H^+^/K^+^-ATPase and AMCase were expressed at much higher levels than those of the housekeeping genes (Fig. 2). The expression of pepsinogen A and C mRNAs were ~300 times and ~15 times higher than that of AMCase, respectively (Fig. 2, upper panel). AMCase mRNA level was comparable to H^+^/K^+^-ATPase and 26 times higher than that of glyceraldehyde-3-phosphate dehydrogenase (GAPDH), 5 times higher than β-actin, and hundreds-to-thousands of times higher than other four tested gastric mucosa genes (Fig. 2, lower panel). These results indicate that AMCase is one of the major transcript in the pig stomach.

### Endogenous pepsins degrade soluble proteins in the stomach extract

**Figure 3:**
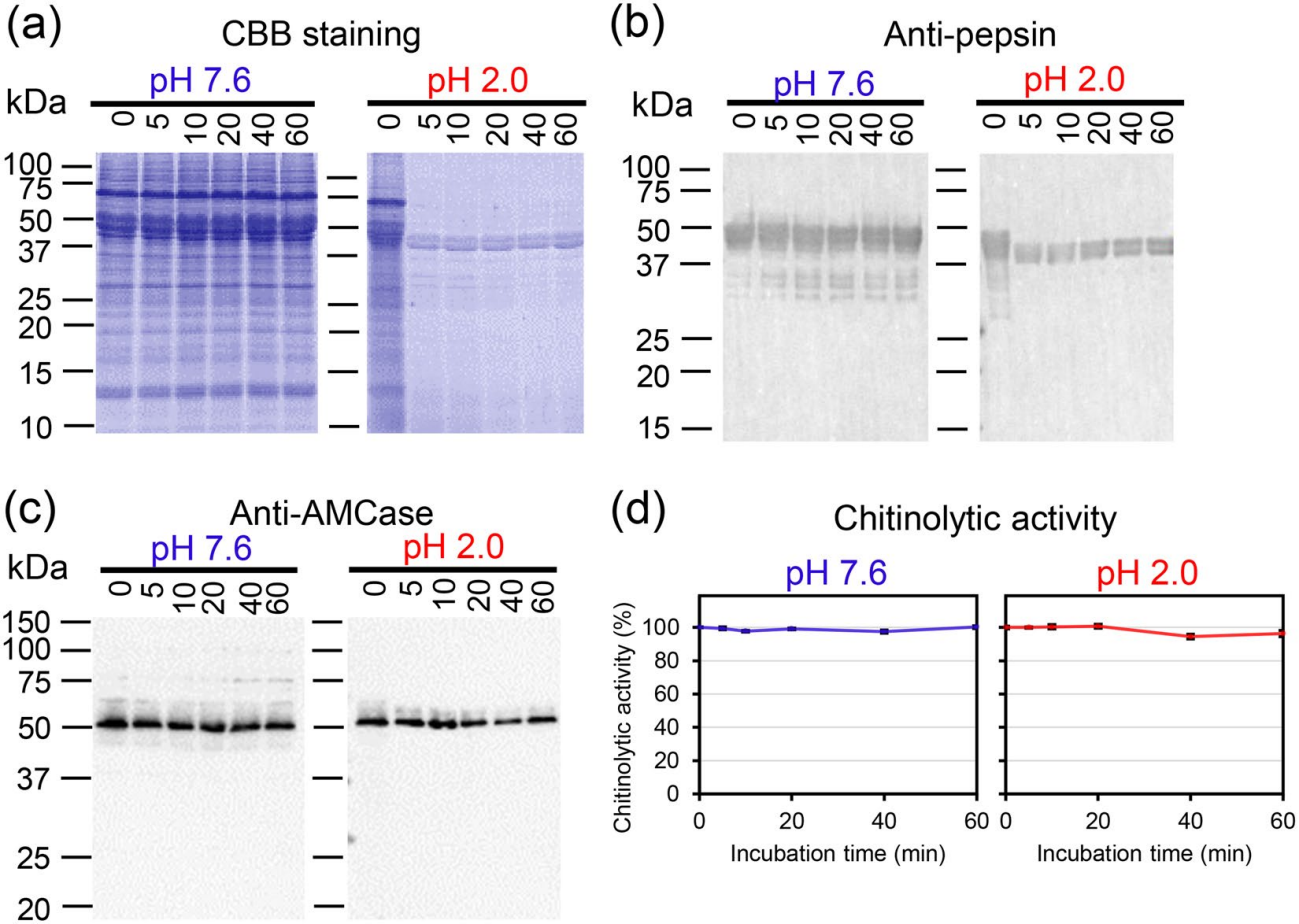
Pepsinogens were converted into active forms and degraded soluble proteins in stomach. Analysis of the endogenous pepsins protease activity in artificially created pig stomach environment (pH 2.0, 37 °C). Soluble protein fractions were prepared from pig stomach tissue in the absence of protease inhibitor and incubated at pH 7.6 or pH 2.0 for up to 60 min and analysed by (**a**) SDS-PAGE and CBB staining. Full-length gel image is shown in Supplementary Fig. S2. (**b**) Western blotting using anti-pepsin or (**c**) AMCase protein using anti-AMCase in the soluble proteins from pig tissue incubated and conducted as described in (**a**). Full-length blots are shown in Supplementary Fig. S3. (**d**) AMCase chitinolytic activity in the extract incubated at pH 7.6 or pH 2.0.

Next, we investigated the protease activity of endogenous pepsins in artificially created pig stomach environment at pH 2.0 and 37 °C. Soluble protein fraction was prepared from the pig stomach in the absence of protease inhibitor and incubated at pH 7.6 or pH 2.0 for up to 60 min. The protein fractions were analyzed by SDS-polyacrylamide gel electrophoresis (PAGE), followed by Coomassie Brilliant Blue (CBB) staining (Fig. 3a). At pH 7.6, no changes in the band pattern and intensities were noticed within 60 min incubation (Fig. 3a). In contrast, we observed time-dependent decrease of the soluble proteins with a marked reduction after as early as 5 min of incubation at pH 2.0, although several bands remained unchanged after 60 min of incubation (Fig. 3a). Western blot analysis using anti-pepsin antibody showed a shift of the respective bands within 5 min of incubation at pH 2.0, indicating pepsinogen-to-pepsin conversion (Fig. 3b).

Western blot analysis using anti-mouse AMCase showed that this enzyme was stable during the 60-min incubation at both pH 7.6 and 2.0 (Fig. 3c). Moreover, the chitinolytic activity measured using 4-nitrophenyl *N,N′*-diacetyl-β-D-chitobioside (4-NP-chitobioside) also remained virtually unchanged (Fig. 3d). These results indicated that AMCase has chitinolytic activity even in the presence of large amount of endogenous pepsins in the pig stomach extract.

### Purification and characterization of pig AMCase from stomach tissue

**Figure 4:**
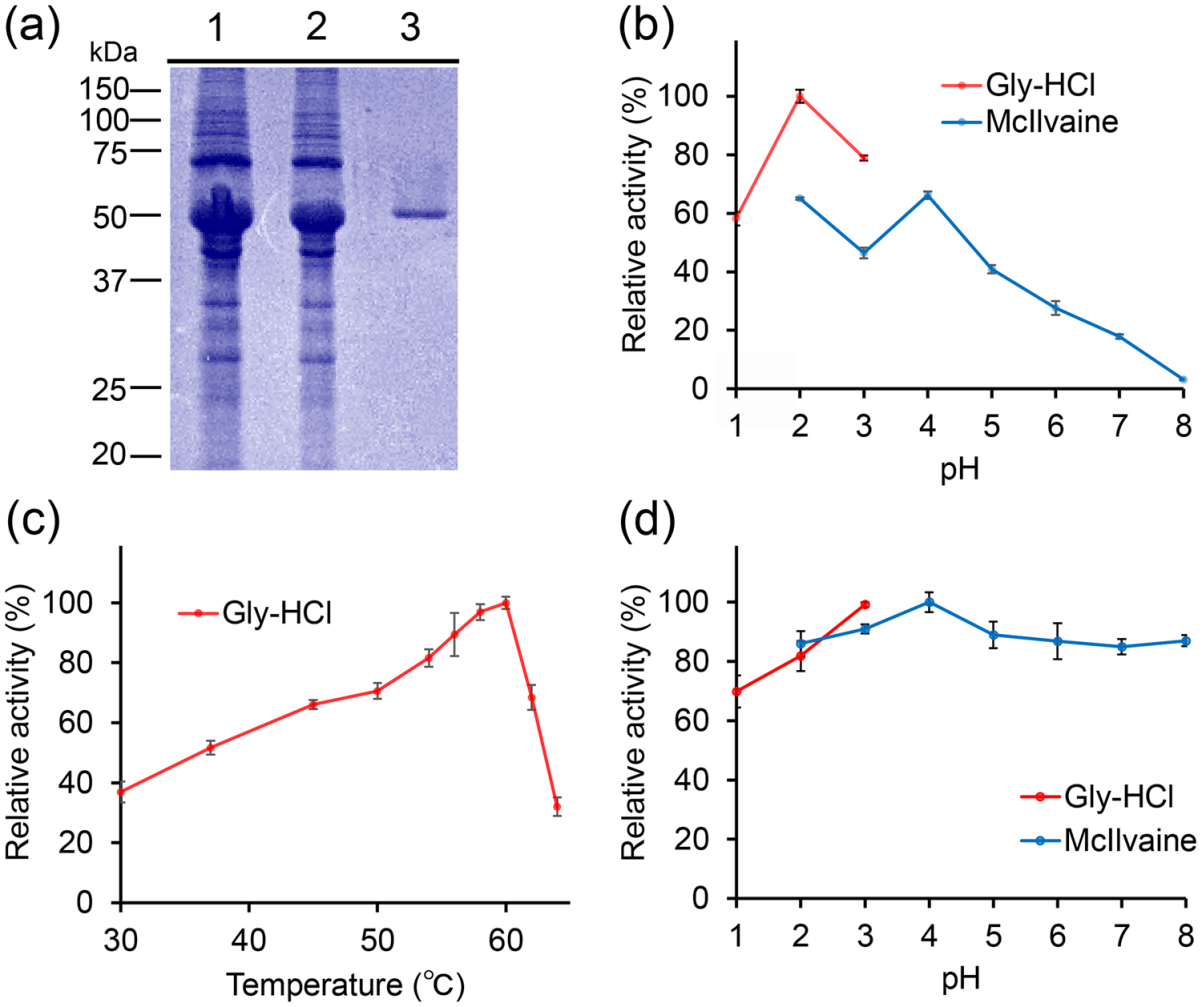
Purification and characterization of AMCase from pig stomach. (**a**) AMCase was purified from pig stomach tissues using chitin beads chromatography as described in the Methods and analyzed by SDS-PAGE and visualized by Coomassie Brilliant Blue (CBB) staining. 1, extract; 2, flow-through; 3, purified enzyme. Full-length gel is shown in Supplementary Fig. S4. (**b**) Optimal pH, (**c**) Optimal temperature and (**d**) pH stability for AMCase activity. Values in (**b**), (**c**,**d**) represent mean ± SD conducted in triplicate.

For further characterization, we purified AMCase by application of the stomach extract onto the chitin beads column. Bound chitinase was eluted from the column with 8 M urea, subsequently removed from the sample as described in Methods. SDS-PAGE analysis of the protein fractions showed one major band at 54 kDa (Fig. 4a). Thus, we obtained purified AMCase usable for *in vitro* enzymatic assays. Purification of the enzyme is summarized in Table 1.

**Table 1:** Purification of AMCase from pig stomach.

Purification step	Total activity (mU)	Total Protein (mg)	Specific activity (mU/mg)	Yield (%)
Total soluble fraction	542.9 ± 3.3	20.2 ± 1.5	26.8 ± 0.2	100
Chitin beads Flow through	61.5 ± 2.4	12.2 ± 2.1	5.0 ± 0.2	11.3
Purified enzyme	49.6 ± 2.1	0.023 ± 0.006	2174.0 ± 90.4	10.3

The purified protein was prepared from 1 G of stomach tissue as described in the Methods.

The pH optima were determined by monitoring enzyme activity at different pH in 0.1 M Gly-HCl (pH 1.0–3.0) or McIlvaine’s (pH 2.0–8.0) buffers using 4-NP-chitobioside as a substrate for 30 min at 37 °C. Highest activity was detected at pH 2.0 in 0.1 M Gly-HCl buffer. Using McIlvaine’s buffer, higher enzymatic activity was observed at pH 2.0–5.0 with peaks at pH 2.0 and pH 4.0 with gradual decrease in less acidic environments (pH 6.0–8.0) (Fig. 4b). Thus, the chitinolytic activity of AMCase has a slightly different pH-related pattern depending on used buffer.

The effect of temperature on enzyme activity was determined in 0.1 M Gly-HCl buffer at pH 2.0 at temperatures ranging from 30 to 64 °C using same substrate for 30 min. As shown in Fig. 3c, the rate of the AMCase-catalyzed reaction was gradually enhanced with increasing temperature and reached the maximum level at 60 °C, then abruptly declined.

We next determined the pH stability of the pig AMCase. The enzyme was pre-incubated on ice for 60 min at various pH in Gly-HCl or McIlvaine’s buffers. The enzyme activity was then analyzed at 37 °C and pH 2.0. As shown in Fig. 4d, the pig AMCase has a remarkable acid stability as the pre-incubation at pH 2.0 caused no measurable decrease in chitinase activity.

### Pig AMCase degrades polymeric chitin substrates under the stomach condition

**Figure 5:**
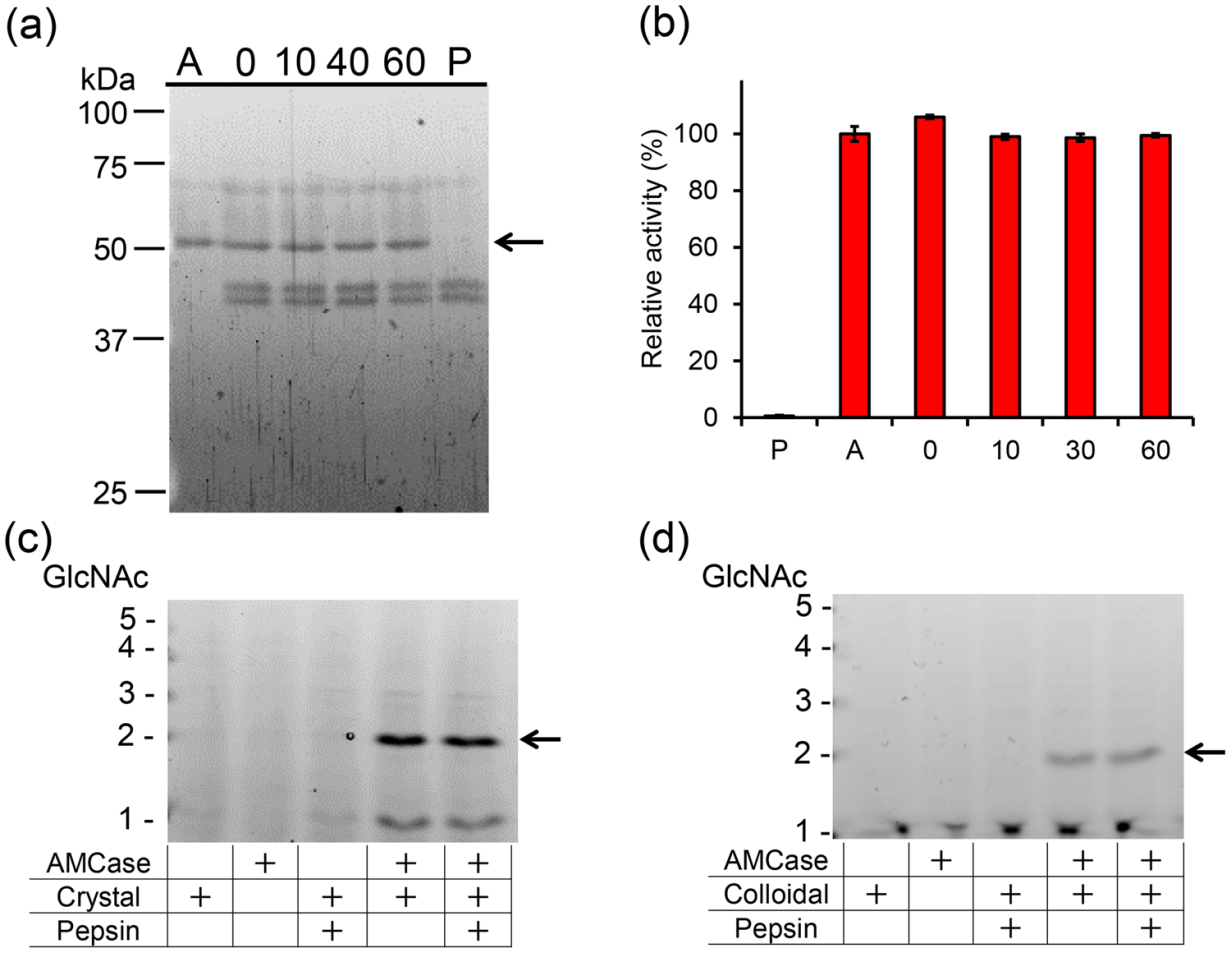
Functional stability of AMCase against pepsin. Purified AMCase was incubated at 37 °C for 0, 10, 40, and 60 min in stomach-like environment in the presence of pepsin. (**a**) Samples were analyzed by SDS-PAGE followed by SYPRO Ruby staining. Full-length gel is shown in Supplementary Fig. S5. (**b**) Determination of the chitinolytic activity. A, AMCase only; P, pepsin only; numbers, incubation time of AMCase and pepsin in minutes. Values in (**b**) represent mean ± SD conducted in triplicate. Degradation products generated by incubation of (**c**) crystalline or (**d**) colloidal chitin with purified enzyme were analyzed by FACE. Full-length gels are shown in Supplementary Fig. S5.

We incubated the purified protein with equal amount of pepsin (0.4 µg) at pH 2.0 for 1 hour and we confirmed the above-observed stability of AMCase at pH 2.0 (Fig. 5a) as well as the maintenance of its chitinolytic activity (Fig. 5b).

Next, we tested whether AMCase can degrade chitin substrates under the pig stomach condition. We incubated colloidal or crystalline chitin with purified AMCase and analyzed the products by an improved fluorophore-assisted carbohydrate electrophoresis (FACE)^37,38^. Purified enzyme degraded both substrates at pH 2.0 as early as after 1 hour incubation and produced primarily (GlcNAc)_2_ fragments in the presence of pepsin (Fig. 5c and d).

### Pig AMCase is resistant to trypsin and chymotrypsin and degrades chitin substrates under intestinal condition

**Figure 6:**
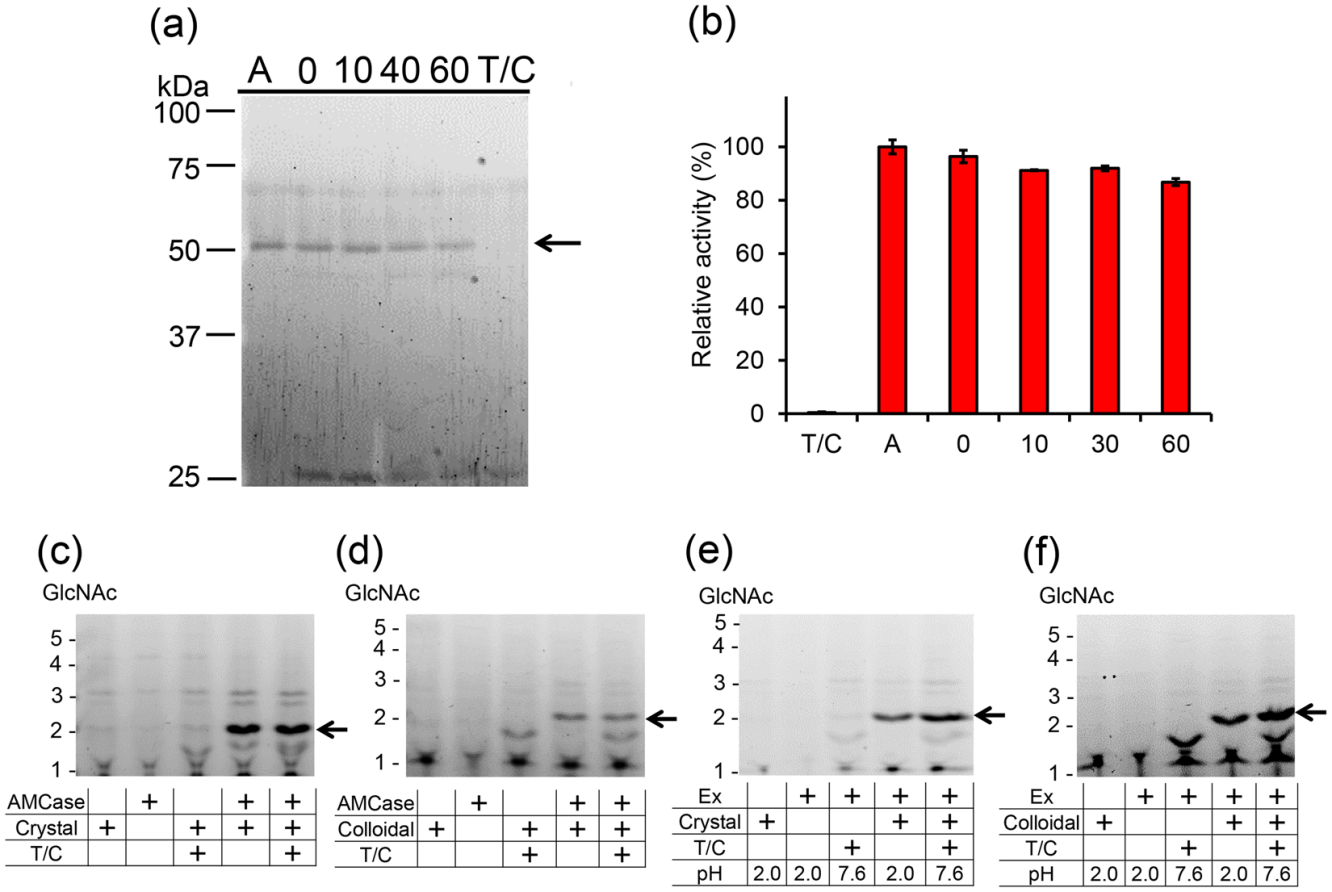
Chitin substrates are degraded by AMCase under gastrointestinal condition. Purified AMCase was incubated at 37 °C for 0, 10, 40, and 60 min under intestine-like environment in the presence of trypsin/chymotrypsin. (**a**) The samples were analyzed by SDS-PAGE followed by SYPRO Ruby staining. Full-length gel is shown in Supplementary Fig. S6. (**b**) Determination of the chitinolytic activity. A, AMCase only; T/C, trypsin/chymotrypsin only; numbers, incubation time of AMCase and trypsin/chymotrypsin in minutes. Values in (**b**) represent mean ± SD conducted in triplicate. Degradation products generated by incubation of **(c**,**e**) crystalline or (**d**,**f**) colloidal chitin with (**c**,**d**) purified enzyme under the intestine condition or with (**e**,**f**) the stomach extract mimicking GIT conditions were analyzed by FACE. Full-length gels are shown in Supplementary Fig. S6.

Next, we investigated whether pig AMCase is also stable under intestinal condition. We incubated the purified protein with equal amount of trypsin/chymotrypsin (0.4 µg) at pH 7.6 and found that AMCase remained stable and active throughout the incubation (Fig. 6a and b).

We incubated crystalline and colloidal chitin with purified AMCase and equal amount of trypsin/chymotrypsin (0.4 µg) at pH 7.6 for 1 hour and the degradation products were analyzed as described above (Fig. 6c and d). Similarly to stomach condition, (GlcNAc)_2_ was produced under the intestine-like condition by AMCase activity. Then, we mimicked the GIT physiology regarding the movement of stomach contents to intestine by pre-incubation of the stomach extract at pH 2.0 for 1 hour, followed by neutralization to pH 7.6 and addition of trypsin/chymotrypsin and further 1-hour incubation (Fig. 6e and f). We observed more (GlcNAc)_2_ degradation products from both colloidal and crystalline chitin after incubation in intestinal environment as compared to single stomach conditions (Fig. 6e and f). Thus, pig AMCase functions as a protease-resistant glycoside hydrolase and can degrade polymeric chitin substrates in both stomach and intestine.

### Chitin in mealworm shells and fruit fly wings is degraded by AMCase and pepsin in the stomach extract

**Figure 7:**
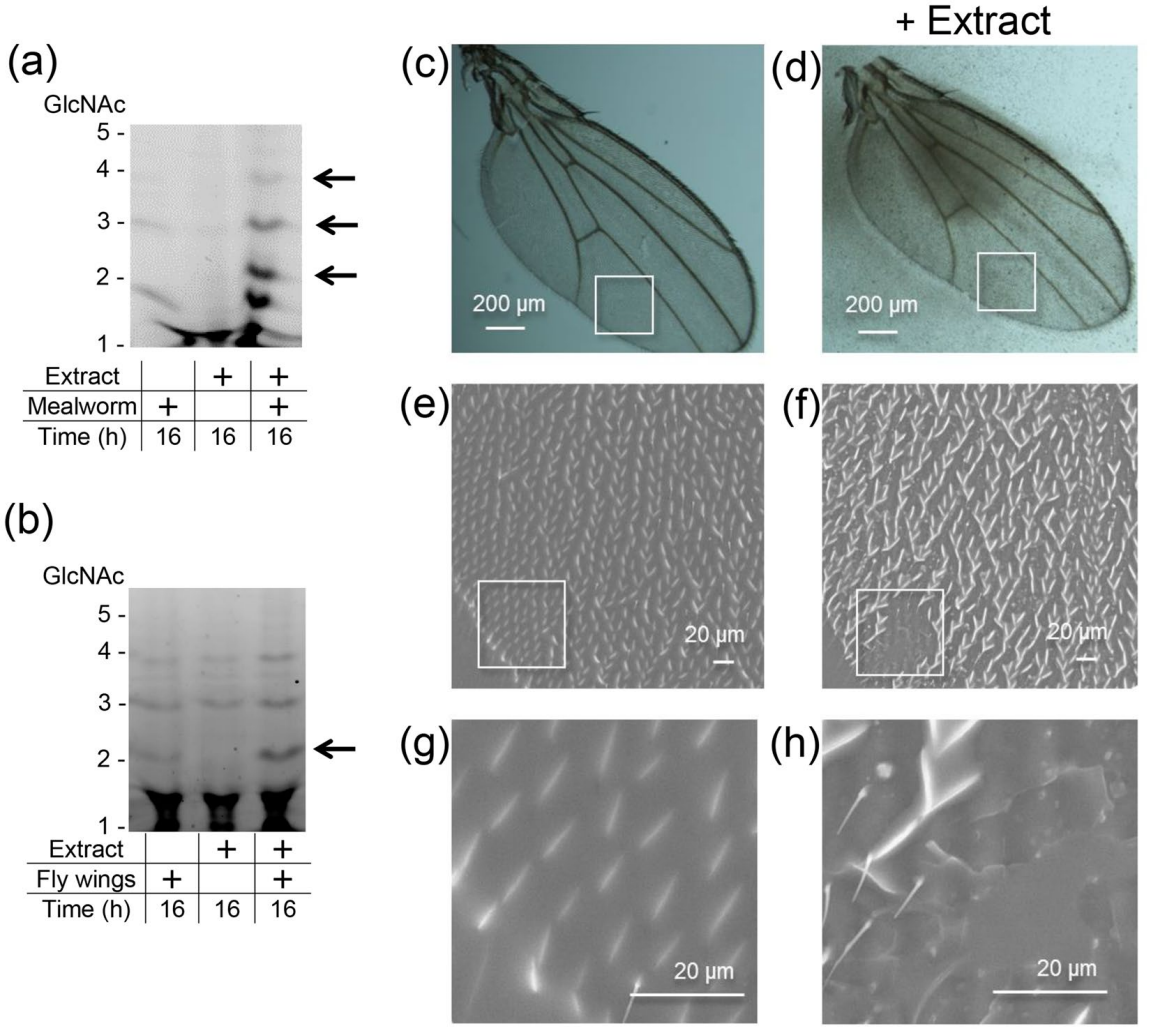
Degradation of mealworm shells and fly wings by stomach extract. Degradation products generated by incubation of (**a**) mealworm shells or (**b**) fruit fly wings with stomach extract were analyzed by FACE as described in the Methods. Full-length gels are shown in Supplementary Fig. S7. Microscopic observations of fruit fly wings incubated with (**d**,**f**,**h**) or without (**c**,**e**,**g**) stomach extract in 0.1 M Gly-HCl (pH 2.0). (**c**,**d**) steromicroscopic photographs. (**e**–**h**) SEM photographs.

Next, we tested whether AMCase and pepsin can degrade chitin-protein substrates present in chitin-containing organism under pig stomach condition. We incubated mealworm (*Tenebrio molitor*) larvae shells with stomach extract at pH 2.0 followed by FACE analysis as described above. AMCase in the stomach extract degraded mealworm shells at pH 2.0 after 16 hours incubation and produced different GlcNAc fragments with the dimer being most abundant (Fig. 7a).

To gain further insights into the digestibility of chitin-containing organisms, we next tested fruit fly (*Drosophila melanogaster Oregon-R*) wings. We first homogenized wings and incubated with the stomach extract and analyzed the products by FACE. We detected (GlcNAc)_2_ fragments (Fig. 7b).

We also visualized the fruit fly wings surface using stereo microscope (Fig. 7c and d) and scanning electron microscope (SEM) (Fig. 7e–h) after 16 hours treatment with (Fig. 7d,f and h) or without (Fig. 7c,e and g) stomach extract in 0.1 M Gly-HCl (pH 2.0). The extract-treated fruit fly wings and the solution became hazy containing some particles (Fig. 7d) and we observed partially damaged regions (Fig. 7f and h). We detected no such damage in wings incubated only with the buffer (Fig. 7c,e and g). These results indicate that AMCase in the stomach extract can deteriorate chitinous fruit fly wing integrity.

## Discussion

Mammalian chitinases have extensively been studied mainly in mice and humans, however relatively little is known about the enzymes in other mammals. In this report, we showed that AMCase mRNA was predominantly expressed in pig stomach tissue and it was much higher than Chit1, housekeeping genes and gastric mucosal genes. We purified AMCase from pig stomach and determined its optimal activity at pH 2.0–4.0 and 60 °C. This enzyme degraded polymeric chitin substrates including mealworm shells and fruit fly wings in the environment mimicking pig GIT conditions. These results support our hypothesis on AMCase functioning as a protease-resistant glycoside hydrolase in the pig digestive system.

AMCase mRNA level was comparable with of H^+^/K^+^-ATPase (role in maintaining the stomach acidity)^39^, and it was substantially higher than mRNA of two housekeeping genes and other gastric mucosa proteins in the pig stomach. This is in agreement with our previous report on high AMCase expression in mouse stomach^32,35^. These data suggest that AMCase in these animals might be able to digest chitin in those animal bodies.

It has been reported that AMCase expression is elevated under several pathological conditions including dry eye syndrome^20,21^. Our qPCR analysis showed that AMCase mRNA expression was ten times lower than that of Chit1 in healthy pig eye. These data imply that AMCase may be a key mediator of innate immune responses in certain ocular pathologies. Chit1 mRNA, on the other hand, is constitutively expressed in healthy mouse eye^32^, human lacrimal gland^40^ and pig eye. Lysozyme is thought to have anti-bacterial effects, whereas Chit1 protects against fungi. Thus, Chit1 probably protects mammalian eyes from chitin-containing pathogens such as fungi, whose cell wall contains high levels of chitin.

Pig pepsin A and C have been purified, crystallized and extensively studied, in the past^41–45^. However, to our knowledge, multiple comparisons of pepsin with other mucosal genes and reference genes mRNA levels have not been performed. Here, we show that pepsinogen A mRNA level was substantially higher than housekeeping genes and gastric mucosa genes in the stomach while exceeding 20 times that the level of pepsinogen C. Although the functional difference between pepsin A and C is still unclear, our results suggest that pepsin A is a major protease and pepsin C acts as a co-protease in the pig stomach.

Pig AMCase was more active in Gly-HCl buffer than in McIlvaine at pH 2.0 (Fig. 4b). Thus, the chitinolytic activity of AMCase has a slightly different pH-related pattern depending on used buffer. Similar results were also obtained in mouse AMCase^35^ and chicken Chia^36^, although with less significant difference in chicken Chia between the two buffers. The reason is not well understood, but some lessons could be learned from human pancreatic α-amylase, whose activation has been shown to be catalyzed by chloride ion^46^. Some members of the amylase protein family require chloride for maximal activity^47–49^. Hydrochloric acid is secreted in the stomach, creating acidic conditions (pH ~2), which may induce similar activation of AMCase. This assumption warrants further scrutiny.

Murine AMCase has been well studied and its optimal activity has been reported at pH 2.0 with a decrease at less acidic conditions (pH 3.0–7.0)^15,35,38^. Also, chicken Chia (AMCase homologue) was most active at pH 2.0 and it retained at less acidic condition^36^. We analyzed effect of pH on the pig AMCase chitinolytic activity and showed that the activity was highest in pH 2.0–4.0 and remained active at up to pH 7.0. Although the pig AMCase shares 81 to 89% primary sequence homology with mouse and chicken counterparts, those specificities to pH and buffer are different. These species particularities can be attributed to differences during the molecular evolution based on changes in feeding habitat.

In our study, we showed that pig AMCase degraded chitin substrates including shells of mealworm larvae and fruit fly wings as well as crystalline and colloidal chitins in the presence of digestive proteases and produced (GlcNAc)_2_. Accordingly, mouse and pig AMCase as well as chicken Chia mainly produced (GlcNAc)_2_^31,35,36^. In addition, we found that mealworm shells digestion by pig AMCase also resulted in (GlcNAc)_3_ and (GlcNAc)_4_ fragments. The product patterns were slightly different from those resulting from colloidal and crystalline chitin degradation. Similarly, we confirmed formation of longer chitooligosaccharides from mealworm larvae shells by chicken Chia^36^. Importantly, we have recently shown that mouse AMCase catalyzes transglycosylation as well as hydrolysis^50^. Thus, it is feasible to assume that distinct chitooligosaccharides can be produced from partially deacetylated chitin in mealworm shells or transglycosylation by pig AMCase.

Pig is one of the major protein resource for humans worldwide. Increasing demand of meat protein requires more feed resources for the livestock. There have been published several studies reporting on application of insects as a sustainable high protein feed ingredient for pig. For example, Jin *et al*.^51^ showed that supplementation of dried mealworm in weaning pigs’ diet improves their growth performance and nutrient digestibility without any detrimental effect on immune responses^51^. Furthermore, chitin derivatives can enhance the immune response and act as an antibiotic/probiotic in pregnant pig^52^. Various biological activities, and especially anti-cancer and anti-inflammatory action of chitin oligosaccharide and chitosan oligosaccharide, have been well studied^9,53–55^. Distinct chitooligosaccharides from chitin-containing organisms may improve immune systems or act as probiotics providing benefits for animal health. Therefore, further evaluation of nutrient value, digestibility and potential side-effects of chitin-containing organisms used as feed ingredient on pig growth performance is needed.

In this report, we showed that AMCase mRNA was highly expressed in pig stomach, having a remarkable protease resistance and degraded chitin or chitin-containing organisms into (GlcNAc)_2_ and several chitooligosaccharides under the GIT condition. We previously reported that similar properties of AMCase and Chia were found in mouse^35^ and chicken^36^, respectively. These animals primarily feed on chitin-containing organisms such as insects and fungi. It is plausible to suggest that their food habitat leads to high expression levels of these enzymes in the stomach and their intense chitinolytic activity in the GIT. According to recent knowledge, chitin-containing organisms can be used as good energy source in pig, chicken and mouse organisms because proteolytic enzyme accessibility is improved by degrading chitinous cuticles of insects and crustaceans by respective AMCase enzymes. From practical point of view, we need further research in other animal species including those with limited ingestion of chitin-containing organisms to reveal whether chitinous diets could be implemented in such species including cattle, sheep, horse, dog, etc.

## Methods

### Pig stomach tissues

Six months-old male pig stomach tissues (Landrace F1) were purchased from Funakoshi Co., Ltd (Tokyo, Japan), which were dissected from the animals, quickly frozen on dry ice and kept at −80 °C.

### RNA and cDNA preparation

Pig Total RNA Panel (Zyagen, San Diego, CA, USA) was used to examine the distribution of transcripts in various tissues. In addition, total RNA was isolated from the pig stomach tissues using TRIzol Reagent (Thermo Fisher Scientific, Waltham, MA, USA) per manufacturer’s instructions and reverse transcribed into cDNA essentially as described previously^32,35,36^.

### Selection of primer pairs for qPCR

Primers for qPCR were designed using PrimerQuest Input (Integrated DNA Technologies, Coralville, IA, USA) and their suitability was evaluated based on a single product generation, as reflected by a single melting temperature as describe previously^32,33,36^. The primers’ sequences are listed in Supplementary Table S1.

### Construction of the DNA standard and qPCR

Construction of the 10 genes standard DNA coding sequences of AMCase, pepsinogen A, pepsinogen C, H^+^/K^+^-ATPase, gastrin, gastric intrinsic factor and mucin were commercially synthesized and inserted into pTAKN-2 vector (Eurofins Genomics, Tokyo, Japan). The standard DNA (956 bases; see Supplementary Fig. S1) was prepared by PCR reamplification from the plasmid DNA using the forward primer 5′-TTGCCGTCCGTGCATATT-3′ and the reverse primer 5′-CAAGGTCAAGGCCATCAAA-3′ and was thereafter used as the standard DNA for qPCR. Each reaction was performed in triplicate.

### Pig stomach extract preparation

Soluble fraction was prepared from pig stomach tissues (0.2 g) by homogenization followed by centrifugation at 15,000 g for 10 min at 4 °C^35,36^. The supernatants were used as the stomach extract and pre-incubated at 37 °C for 0, 10, 20, 40 or 60 min at pH 7.6 or pH 2.0. After incubation at pH 2.0 and 37 °C, the solutions were neutralized.

### SDS-polyacrylamide gel electrophoresis and Western blot

The obtained protein fractions were analyzed using standard SDS-PAGE, followed by Coomassie Brilliant Blue R-250 (CBB, Sigma-Aldrich, St. Louis, MO, USA) or Western blot using polyclonal anti-mouse C-terminal AMCase^33^ or polyclonal pig anti-pepsin antibody (GeneTex, Irvine, CA, USA), followed by peroxidase-conjugated AffiniPure F (ab’)_2_ Fragment Donkey Anti-Rabbit IgG (H + L) (Jackson ImmunoResearch Laboratories, Inc., West Grove, PA, USA) or AffiniPure Donkey Anti-Goat IgG-HRP (Jackson ImmunoResearch laboratories). The immunoblots were analyzed and quantified by Luminescent Image Analyzer (ImageQuant LAS 4000, GE Healthcare, Piscataway, NJ, USA) according to the manufacturer’s instructions.

### Chitinase enzymatic assays

Chitinolytic activity was determined using a synthetic chromogenic substrate, 4-nitrophenyl *N,N*′-diacetyl-β-D-chitobioside (4-NP-chitobioside, Sigma-Aldrich) essentially as described previously^31^. All enzymatic reactions for optimum pH and temperature determination of were conducted in a volume of 50 μL as described previously^31,56^.

### Purification of pig AMCase

AMCase was purified from pig stomach tissue (1 g) using chitin beads column and eluted with 8 M urea as performed previously^36^. Protein concentrations were determined by the Bradford Protein Assay (Bio-Rad Laboratories, Hercules, CA, USA) using the BioPhotometer Plus UV/Vis photometer (Eppendorf, Hamburg, Germany), with bovine serum albumin as a standard. AMCase unit definition was also reported previously^31^.

### The effects of pH and temperature on chitinase activity

For determination of the optimal pH, the chitinase activity was investigated by incubating the enzyme with 4-NP-chitobioside as a substrate in 0.1 M Gly-HCl buffer (pH 1.0–3.0) or McIlvaine’s buffer (0.1 M citric acid and 0.2 M Na_2_HPO_4_; pH 2.0–8.0) at 37 °C for 30 min. To measure the optimal temperature, chitinase activity was assayed between 30 °C and 64 °C in 0.1 M Gly-HCl buffer (pH 2.0).

For the determination of the pH stability, samples were incubated for 1 hour on ice in 0.1 M Gly-HCl buffer (pH 1.0–3.0) or McIlvaine’s buffer (pH 2.0–8.0). After the pre-incubation at the indicated pH, the residual activity was analyzed at pH 2.0 in 0.1 M Gly-HCl buffer, as described above.

### Mealworms

Jumbo mealworm (*Tenebrio molitor*) larvae were purchased from local commercial supplier (Lumberjack Co., Ltd., Tokyo, Japan). We used the shells containing connective tissue as chitin-protein polymer substrates as described previously^36^.

### Degradation of colloidal and crystalline chitin substrates and mealworm larvae shell

Colloidal and crystalline chitin were incubated in a volume of 50 µL containing purified AMCase (4 mU) or soluble protein (4 mU) from pig stomach as described previously^35,36^. Mealworm larvae shells were also incubated with soluble protein fraction (20 mU) in an analogous way. Generated chitin fragments were analyzed by fluorophore-assisted carbohydrate electrophoresis (FACE) as originally described by Jackson^37^ and recently improved by us^38^.

### Chitin degradation of fruit fly wings by stomach extract

Fruit flies (*D. melanogaster Oregon-R*) were bred at the facility in Okayama University. Flies were immersed once in ethanol. Fifty wings were homogenized using TaKaRa BioMasher Standard (TaKaRa Bio, Shiga, Japan) and then treated with stomach extract as described in mealworm. After incubation at 37 °C for 16 hours, degradation products were analyzed by FACE as described above. For morphological examination, wing was treated with stomach extract containing 9 mU AMCase activity in 0.1 M Gly-HCl (pH 2.0) at final volume of 10 µL using a glass slide printed with water-repellent mark (TF1205, Matsunami Glass Ind., Ltd., Osaka, Japan). The morphological changes of the wings were assessed using a stereo microscope (M205 C, Leica Microsystems, Wetzlar, Germany). Observations of fly wings by scanning electron microscope (SEM, JCM-6000, acceleration voltage: 15 kV, JEOL Ltd., Tokyo, Japan) were achieved by ionic liquids coating method without accumulation of electron charges, indicating that the liquid behaves as an electronically conducting material^57^. Observed sample was immersed into mixture of ionic liquid, 10% 1-hexyl-3-methylimidazolium bis (fluorosulfonyl) imide (Mitsubishi Materials Electronic Chemicals Co., Ltd., Akita, Japan) in ethanol. Immersed fly wing samples were dried at room temperature over 1 hour prior to SEM observations for removing ethanol. Thin and uniform ionic liquid coating layer enabled clear SEM observation without charge up of surface of measurement samples.

### Statistical analysis

Biochemical data were compared by Student’s t-test. We carried out experiments in triplicate for the statistical analysis

## Electronic supplementary material

Supporting information
