# Supplementary material for: Protease resistance of porcine acidic mammalian chitinase under gastrointestinal conditions implies that chitin-containing organisms can be sustainable dietary resources
Source: Sci Rep. 2017 Oct 11;7:12963. doi: 10.1038/s41598-017-13526-6 (PMC5636921; doi:10.1038/s41598-017-13526-6)
Supplement: Supplementary file 1 — Supporting information [file 41598_2017_13526_MOESM1_ESM.pdf]

Supplementary Information

Protease resistance of porcine acidic mammalian chitinase under gastrointestinal conditions implies that chitin-containing organisms can be sustainable dietary resources

Eri Tabata[1], Akinori Kashimura[1], Satoshi Wakita[1], Misa Ohno[1], Masayoshi Sakaguchi[1], Yasusato Sugahara[1], Yasutada Imamura[1], Shiro Seki[2], Hitoshi Ueda[3], Vaclav Matoska[4], Peter O. Bauer[4, 5], Fumitaka Oyama[1]

[1]Department of Chemistry and Life Science, Kogakuin University, Hachioji, Tokyo 192-0015, Japan, [2]Department of Environmental Chemistry, Kogakuin University, Hachioji, Tokyo 192-0015, Japan, [3]Department of Integrative Biology, Graduate School of Natural Science and Technology, Okayama University, Okayama 700-8530, Japan, [4]Laboratory of Molecular Diagnostics, Department of Clinical Biochemistry, Hematology and Immunology, Homolka Hospital, Roentgenova 37/2, Prague 150 00, Czech Republic, [5]Bioinova Ltd., Videnska 1083, Prague 142 20, Czech Republic

(a)

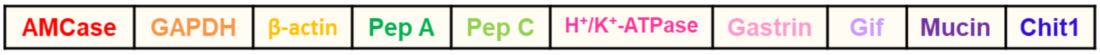

(b)

TGACTTCACAGGCACTTTCTGCAACCAGGGCAAGTTCCCCCTGATCAACACCCTGAAGGATGCT
CTTGGCCT**GAATAGCACAAGTTGCACCGACCTCCACTACATGGTCTACA**TGTTCCAGTATGATT
CCACCCACGGCAAGTTCCACGGCACAGTCAAGGCG**GAGAACGGGAAGCTTGTCATCTTCTAGGC**
GGACTGTTAGTTGCATTCCACCTTTTTCTTGACAAAACCTAACTTGCGCAGAAAAT**GAGATGAG**
ATTGGCATGGCT*GGATCC***GGCGGCATCGATTCTTCTTA**CTACACAGGAAGCCTGAACTGGGTGC
CTGTTTCCGTCGAGGGTTACTGGCAG**ATTACCTTGGACAGCATCACCGAGTTCCTCAAGACCCA**
CAAGTACGACCCCGCCCAGAGGTACCGCTTCGGCGACTTCAGCGTGGCCTTGGAGCCCATGGCC
TACTTGGAAGCTGCC**TACTTTGGCGAGATCAGCATCCTGGGTTCACTGACTACTTCAC**GGCCAT
GGCCCAGGAGGGCTGGTTCCCACTGCTGTGTGGGGCTGCGGCCACAGTGG**GAGAACCACCAC**
CTACAAGACGGACCGGTGGTCTCTTCTATTAACAATATCGCTGAAAATGTCAACCACAGGACGT
ACTGGCAGTTTCTGAGTGGCCAAACGCC**CTTAAACGAAGGAGTTGCGGAATGGATGGAGGAGGA**
AGAAGAAGCATATGGATGGATGGACTTCGGCCGCCGCAGTGCTGAGGAAGGAGACCAGCGTC**CC**
TAGAACCGAGCTCCAGACCACAACCTGAAGACACAGTTTGATCGGCTTAAAGCGGAAGCAGGCA
CCTATAACCTGACCATCAGTGAAGTTAGTGTGATTGATGCACC**ATTTCCTTCCTCTGCTCAGC***G*
*GATCC***GTCAACTCAGCCATCAGGTT**TCTGCGCAAATATGGC**TTTGATGGCCTTGACCTTG**

Figure S1. Single standard DNA molecule used for qPCR. (a) Schematic representation of the standard DNA molecule. (b) Nucleotide sequence of the single standard DNA. The single standard DNA, 956 bases long, contained cDNA fragments of AMCase, GAPDH, β-actin, pepsinogen A (Pep A), pepsinogen C (Pep C), H^+/K^+-ATPase, Gastrin, gastric intrinsic factor (Gif), Mucin and Chit1 in a one-to-one ratio. Primers for qPCR in each target DNA region are shown in underline.

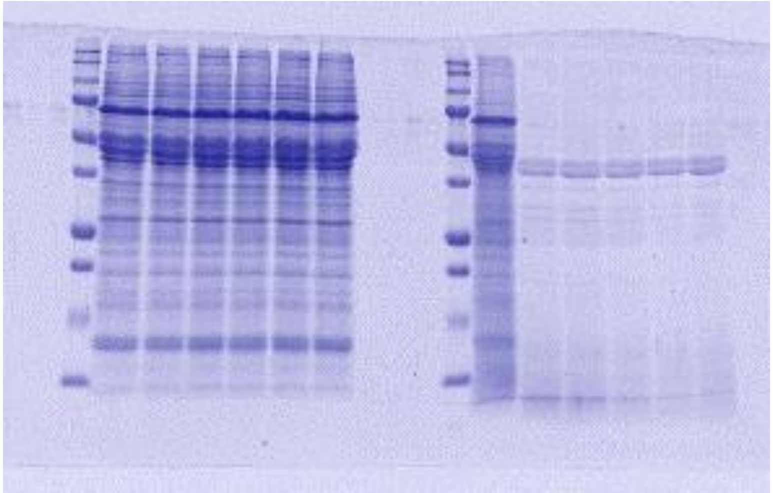

Figure S2. Full-length gel image shown in Fig. 3a. Full-length gel images of SDS-PAGE and CBB staining.

(a)

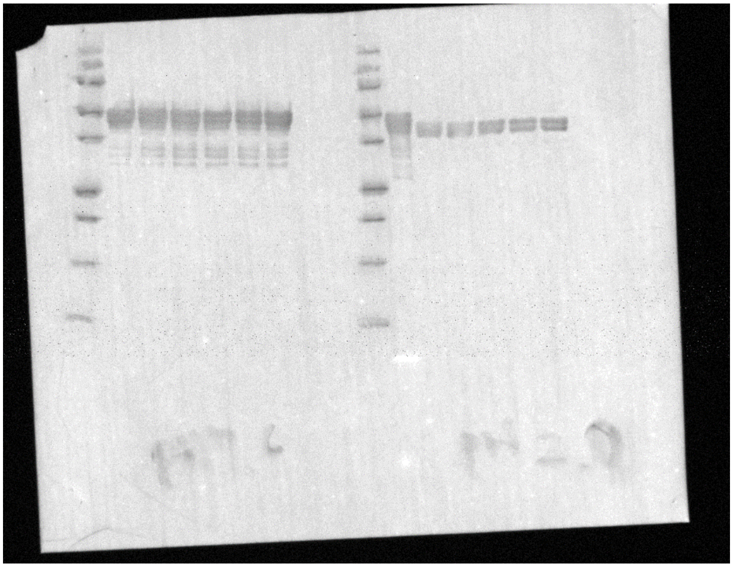

(b)

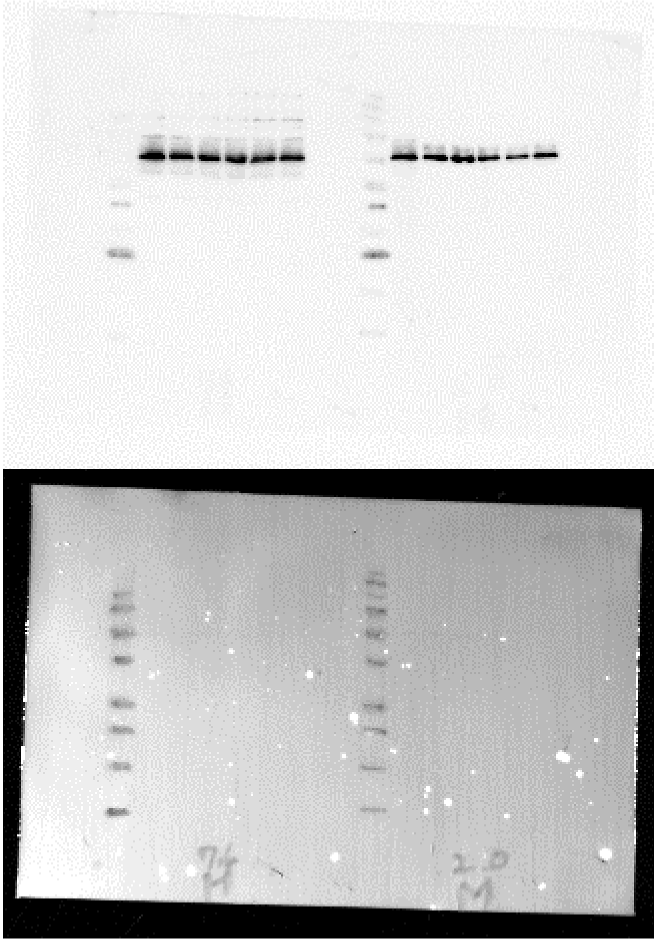

Figure S3. Full-length gel and blots shown in Fig. 3b and c. (a) Western blotting using anti-pepsin and molecular weight markers. (b) Western blotting using anti-AMCase and its molecular weight markers.

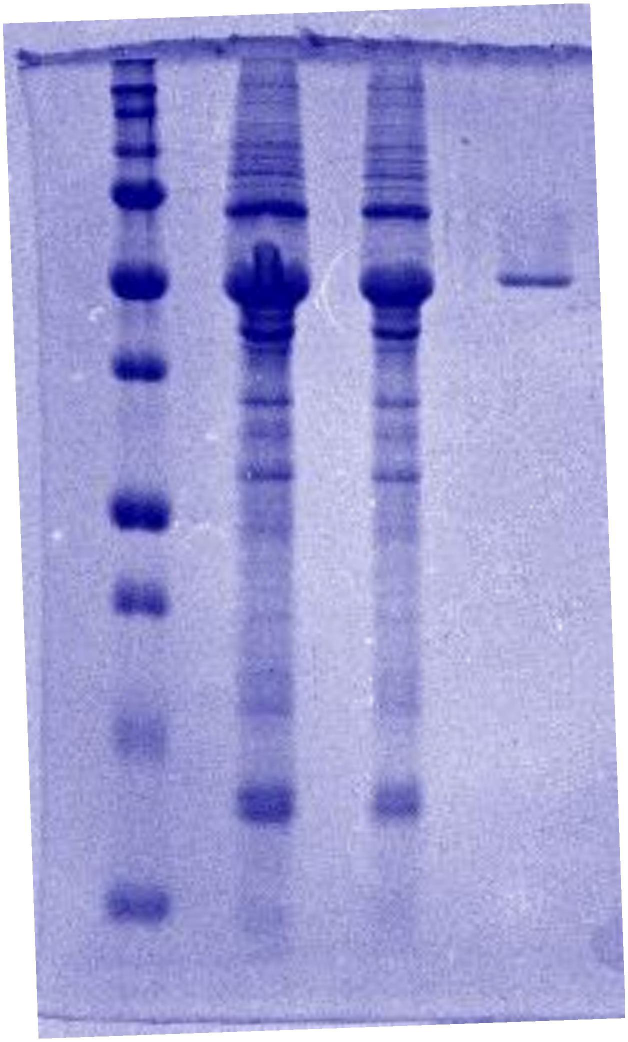

Figure S4. Full-length gel image shown in Fig. 4. Full-length gel image of SDS-PAGE and CBB staining.

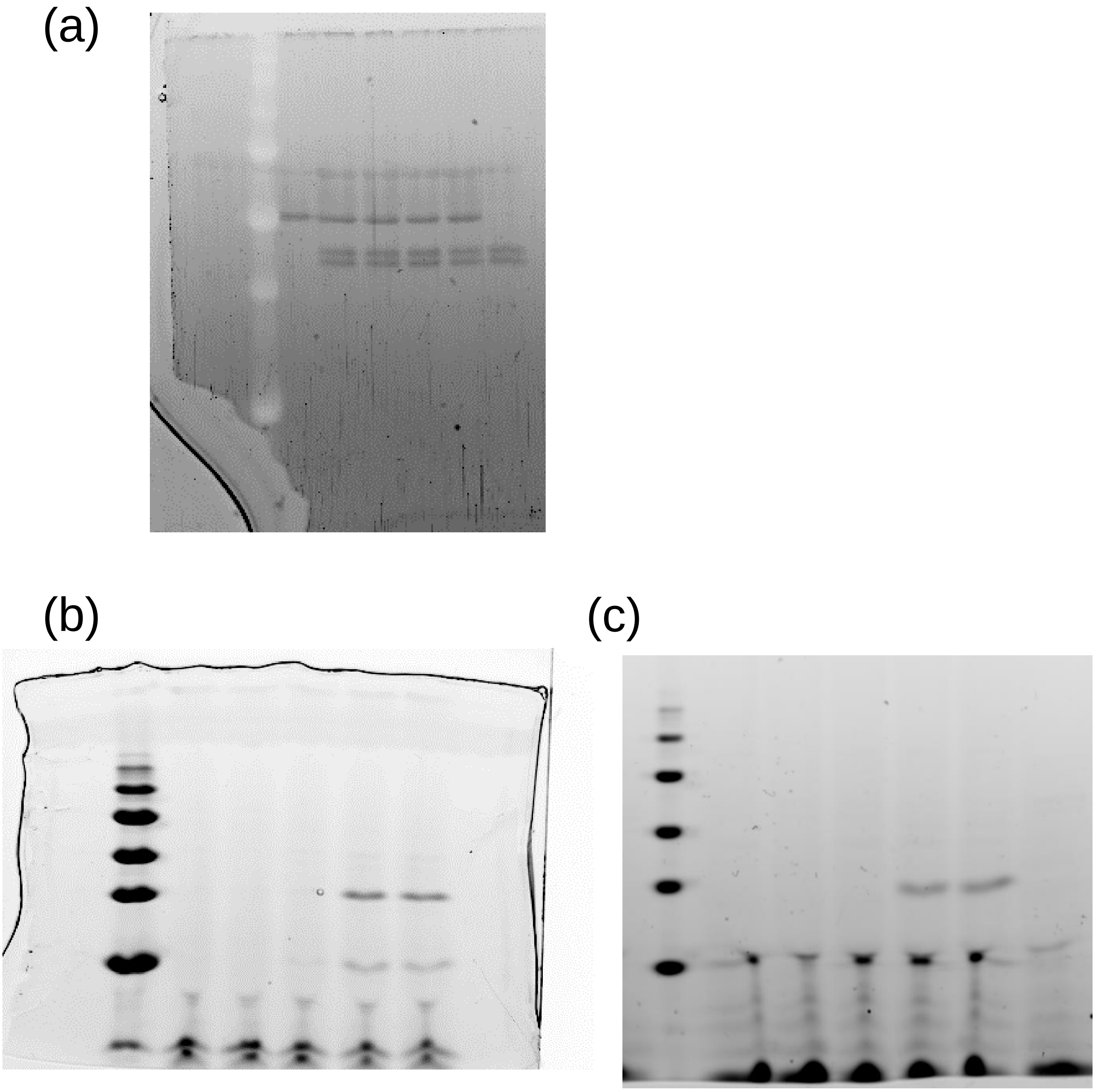

Figure S5. Full-length gel and blots shown in Fig. 5. (a) Full-length gel images of SDS-PAGE and SYPRO Ruby staining in Fig. 5a. (b and c) Full-length gel images of FACE methods in Fig. 5c and d.

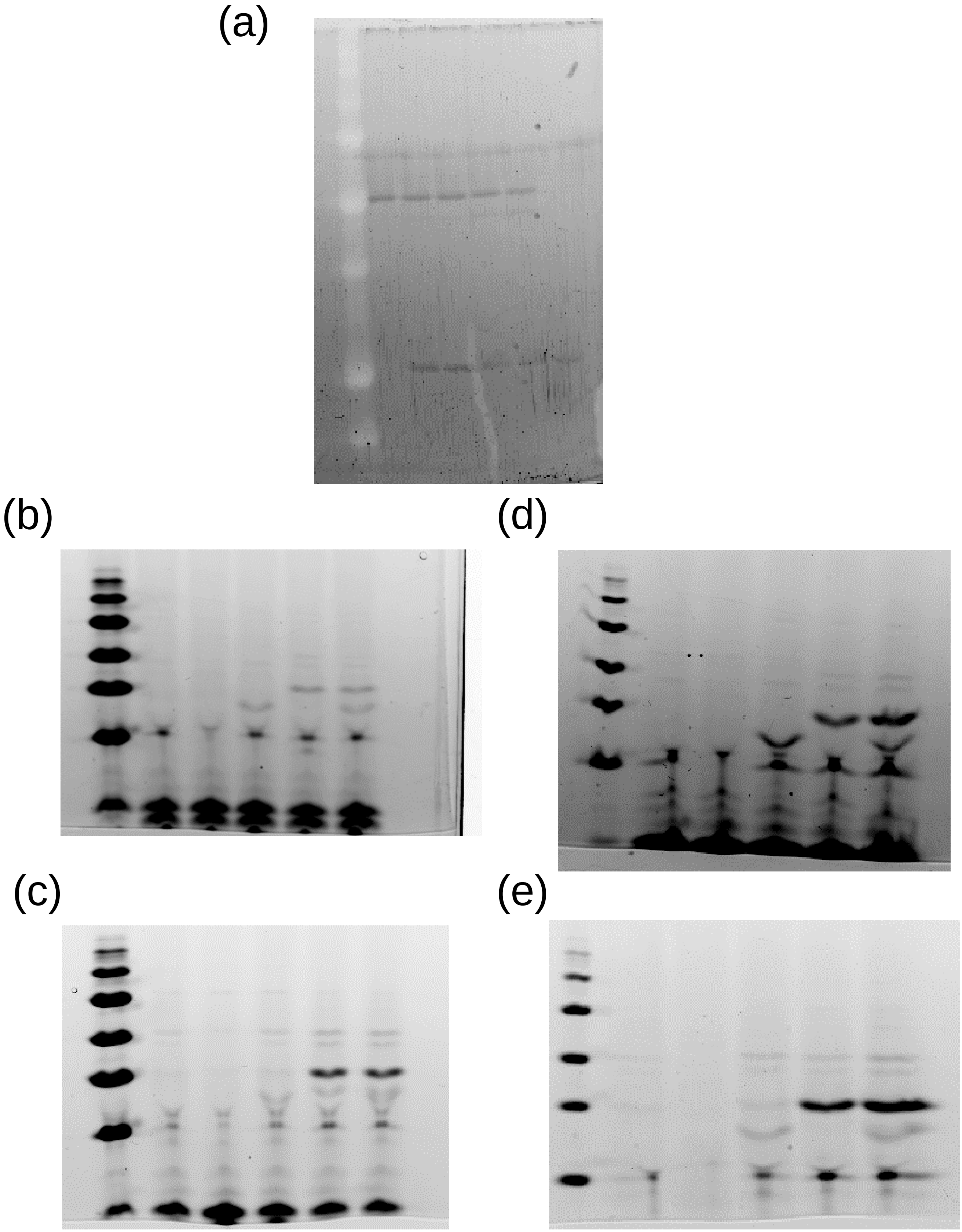

Figure S6. Full-length gel and blots shown in Fig. 6. (a) Full-length gel images of SDS-PAGE and SYPRO Ruby staining in Fig. 6a. (b-e) Full-length gel images of FACE methods in Fig. 6c-f.

(a) (b)

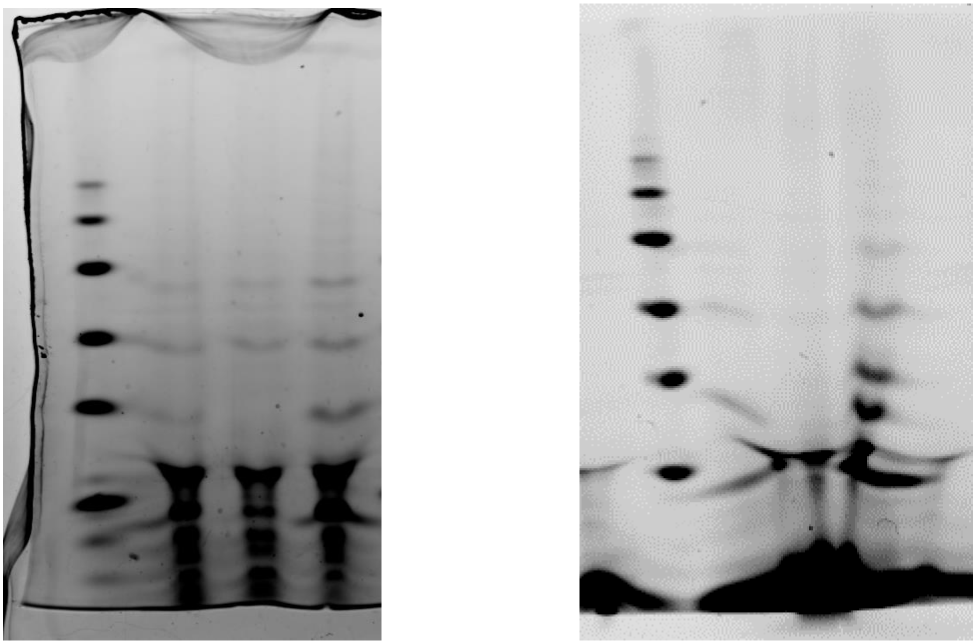

Figure S7. Full-length gel and blots shown in Fig. 7. (a and b) Full-length gel images of FACE methods in Fig. 6a and b.

Table S1. List of qPCR primers.

Gene	Forward primer	Reverse primer
AMCase	TGACTTCACAGGCACTTTCT	CGGTGCAACTTGTGCTATTC
GAPDH	ACCTCCACTACATGGTCTACA	ATGACAAGCTTCCCGTTCTC
β-actin	CTTCTAGGCGGACTGTTAGTTG	AGCCATGCCAATCTCATCTC
Pepsinogen A	GGCGGCATCGATTCTTCTTA	ATTACCTTGGACAGCATCACC
Pepsinogen C	GAGTTCCTCAAGACCCACAAG	GATGCTGATCTCGCCAAAGTA
H^+/K^+-ATPase	CTGGGTTCACTGACTACTTCAC	GAGAACCACCACCTACAAGAC
Gastrin	GGACCGGTGGTCTCTTCTATTA	TCCGCAACTCCTTCGTTTAAG
Gif	ATGGATGGAGGAGGAAGAAGA	TCTGGAGCTCGGTTCTAGG
Mucin	CCACAACCTGAAGACACAGT	GCTGAGCAGAGGAAGGAAAT
Chit1	GTCAACTCAGCCATCAGGTT	CAAGGTCAAGGCCATCAAA
